# Investigating the Conformation of S100β Protein Under Physiological Parameters Using Computational Modeling: A Clue for Rational Drug Design

**DOI:** 10.2174/1874120701812010073

**Published:** 2018-10-17

**Authors:** Elvis K. Tiburu, Ibrahim Issah, Mabel Darko, Robert E. Armah-Sekum, Stephen O. A. Gyampo, Nadia K. Amoateng, Samuel K. Kwofie, Gordon Awandare

**Affiliations:** 1 Department of Biomedical Engineering, College of Basic and Applied Sciences, University of Ghana, P. O. Box LG 25, Legon, Ghana; 2 Department of Biochemistry, Cell and Molecular Biology, University of Ghana, P. O. Box LG 25, Legon, Ghana; 3 West African Centre for Cell Biology of Infectious Pathogens, University of Ghana, P. O. Box LG 25, Legon, Ghana

## Investigating the Conformation of S100β Protein Under Physiological Parameters Using Computational Modeling: A Clue for Rational Drug Design

The Open Biomedical Engineering Journal, **2018**, 12: 36-50

The name of country in affiliations is mentioned below:

**^1^ Department of Biomedical Engineering, College of Basic and Applied Sciences, University of Ghana, P. O. Box LG 25, Legon, Ghana**

**^2^ Department of Biochemistry, Cell and Molecular Biology, University of Ghana, P. O. Box LG 25, Legon, Ghana**

**^3^ West African Centre for Cell Biology of Infectious Pathogens, University of Ghana, P. O. Box LG 25, Legon, Ghana**

The name of country in affiliations provided was: 

**^1^ Department of Biomedical Engineering, College of Basic and Applied Sciences, University of Ghana, P. O. Box LG 25, Legon**

**^2^ Department of Biochemistry, Cell and Molecular Biology, University of Ghana, P. O. Box LG 25, Legon**

**^3^ West African Centre for Cell Biology of Infectious Pathogens, University of Ghana, P. O. Box LG 25, Legon**

